# *eBioMedicine* in 2022: A look back and the mission ahead

**DOI:** 10.1016/j.ebiom.2022.104416

**Published:** 2022-12-14

**Authors:** eBioMedicine

As 2022 concludes, we would like to take this opportunity to look back at some of the most exciting research we published this year. Although we have had the privilege of publishing strong translational papers covering the whole spectrum of human health, we chose to highlight a few articles in three topical areas of particular importance: COVID-19, gut microbiome, and the health effects of climate change.

An impressive number of articles were published on COVID-19 in 2020 and 2021. However, with the circulation of novel variants of concern, the need for vaccines suited for different populations, and long COVID, scientists and clinicians were not lacking new research questions in 2022.

After the development and deployment of the first vaccines (from Pfizer, BioNTech, and Moderna for mRNA types and AstraZeneca and Janssen for adenovirus vector types), there was the question of the optimal vaccination strategy. The HEVACC study group, with a multicentre, partially randomised trial in June, substantiated the hypothesis that a heterologous schedule of vaccination (ie, AstraZeneca and BioNTech) would be safe and more efficient than a homologous one (ie, two doses of AstraZeneca). In November, German teams led by Ulrike Protzer, Kilian Schober, and Matthias Tenbusch supported the advantages of heterologous vaccination by comparing the long term dynamics (up to 180 days) of humoral and cellular immune responses.

To immunise the global population, the safety and immunogenicity of affordable and stable vaccines have been tested in clinical trials. Two such trials were published in our journal. The first one, Corbevax from Biological E, has three key components: an RBD antigen, alum, and CpG1018 adjuvants. This trial was successful and helped find the optimal formulation before moving on to a phase 3 trial. Unfortunately, although the preclinical results of the second vaccine candidate, V590 from Merck, were promising, a low immunogenicity was observed in the phase 1 trial. This vaccine development was then discontinued.

Vaccines that are currently in use provide effective protection against severe COVID-19, but do not completely prevent virus transmission. Intranasal vaccines have been proposed to induce mucosal immunological responses and block pathogens at the entry point. Dennis Christensen and colleagues from the Center for Vaccine Research, Denmark, tested such a strategy in an animal model. Administering an intranasal booster to Syrian hamsters that had previously been injected with a subcutaneous version conferred them protection against SARS-CoV-2 infection and onward transmission, even although the virus could still be detected in the upper airway. In a first-in-human trial, Meera Madhavan and colleagues from Oxford, UK, tested whether administering a booster dose of the AstraZeneca vaccine intranasally would provide similar protection to the intramuscular administration. Although it was well tolerated, this modality did not induce a consistent mucosal antibody response.

Major research efforts are also targeting the post-COVID-19 condition, known as long COVID. The diagnosis of this condition is complex and hindered by the fact that many patients are unsure of their infection history. Benjamin Krishna and colleagues from the University of Cambridge, UK, showed that a T-cell based assay could provide evidence of past infection, even for patients who were SARS-CoV-2 antibody negative, in a cohort of 72 patients. As for all medical conditions, identifying individuals who are most at risk of developing these symptoms could help improve outcomes. Gabriella Captur and colleagues from UK COVIDsortium proposed a plasma proteomics signature with the potential to identify, at the time of infection, individuals most likely to have long COVID up to 1 year later. Their analysis of 156 health-care workers also showed that non-severe infection perturbs the plasma proteome for at least 6 weeks. Unfortunately, the mechanisms underlying this condition remain poorly understood. We published two studies aiming to increase our knowledge of the consequences on brain and cardiac functions of the disease. Christopher Käufer and colleagues from University of Hannover, Germany, observed in an animal model, after apparent recovery, an increase in markers of neuroinflammation and accumulation of proteins that are implicated in the pathogenesis of neurodegenerative diseases. Joyce Lu and colleagues from the Albert Einstein College of Medicine, NY, USA, investigated the incidence of persistent acute cardiac injury and the factors associated with its developments in patients hospitalised at their centre. Acute cardiac injury was observed in 40% of their patients, and 56% of the patients who returned for follow-up 2·5 months after infection exhibited persistent injury. Standard laboratory markers (eg, troponin, creatinine, lymphocyte, sodium, lactate dehydrogenase, and hematocrit) could be used to predict recovery of the patients.

Another topic that attracted a lot of attention from both authors and readers is the gut microbiome and its role in a range of diseases. We published a series of reviews of such new findings discussing the interactions between microbiome, host immunity, and cancer; its effects on anticancer drugs; the link between gut dysbiosis and rheumatic diseases; and the gut–brain axis in the context of neurodegenerative and neurodevelopmental diseases. Furthermore, Yue Ma, Xiaolin Liu, and Jun Wang from the Chinese Academy of Sciences, Beijing, China, provided a detailed review of the shifts in gut microbial metabolites observed in patients with different complex diseases (eg, hepatitis, atherosclerosis, and type 2 diabetes).

Novel research that increased our understanding of the role of gut microbiota in multiple sclerosis and infantile spasms was also published. Claudia Cantoni and colleagues from Washington University, St Louis, USA, did a 6-month, longitudinal, multi-omics study of 24 individuals with multiple sclerosis and 25 control individuals. They identified multisystem alterations in the gut microbiota, the immune system, and the blood metabolome of patients, emphasising the need for an integrated approach to improve treatment and diagnosis of this disease. Kathryn Fitzgerald and colleagues evaluated, in a randomised controlled study, the effects of different calorie-restricted diets in 31 people with multiple sclerosis. They observed that an intermittent calorie-restricted diet was associated with more substantial changes (eg, reduction in lipid biomarkers and in memory T cell subsets) than a chronic energy-restricted diet. As an intermittent diet is also considered to be more feasible than a chronic diet, such an intervention has the potential to be used as a complementary therapeutic approach to enhance the effect of disease-modifying therapies.

Epilepsy is another disease that could benefit from modulation of the gut microbiome via a dietary intervention, namely the ketogenic diet. The team of Jane Shearer and Morris Scantlebury from the University of Calgary, Canada, presented two preclinical studies that provided insights into the optimisation of this treatment of infantile spasms. The first study highlighted the importance of tryptophan-kynurenine metabolism in this condition and suggested new therapeutic approaches. The second study focused on a means to alleviate the side-effects of this intervention. The ketogenic diet can damage the liver, but the authors showed an early intervention with probiotics could alleviate this side-effect. Bringing this intervention closer to clinical relevance, Maria Dahlin and colleagues from Karolinska Hospital, Sweden, identified, in an observational study of 28 children, a signature associated with an anti-epileptic response to the ketogenic diet treatment. This signature could be used to identify potential responders before initiating treatment or to develop novel treatments based on these therapeutic targets.

Finally, a substantial part of our efforts in 2022 was dedicated to the effects of climate and environmental changes on health. Although the reality of climate change is well documented, scientists and funding bodies worldwide (eg, the HERA group in Europe, National Institutes of Health, and the National Science Foundation in the USA) warned that its effects on human health are still poorly understood and proposed research agendas. As a translational journal, *eBioMedicine* has a role to play. Last year, we highlighted the importance of deciphering mechanisms of environment-related health outcomes and the need for new insights for climate-adaptive therapies or mitigation strategies. To support this aim, in 2022 we published several articles on the effects of air pollution on the risk of chronic obstructed pulmonary disease, on cardiometabolic multimorbidity, ovarian response, on the immune system, and susceptibility to pneumococcal infection. The record temperatures observed worldwide this year, and the related increase in mortality risk, highlighted the importance of evaluating mitigation approaches. Hayon Choi and colleagues from 24 countries in Europe, the Americas, and Asia showed that cities with large greenspaces had lower heat-related mortality risk compared with cities with small greenspaces. These findings support previous, smaller studies and encourage communities to develop greenspaces to avoid the urban heat island effect.

According to the European Environment Agency, only about 500 chemicals are extensively characterised for their hazards and exposures; approximately 100,000 others should be studied further. Astrid Sevelsted and colleagues from the University of Copenhagen, Denmark, proposed to fill some of these gaps using data from a population-based cohort of 738 pregnant people and their children who were followed up from 24 weeks of gestation until age 10 years. They detected perfluoroalkyl substances (PFOS and PFOA, persistent and bioaccumulative chemicals) in all samples (both birthing parents and children) of the cohort. Concentrations of these substances were associated with reduced intrauterine growth. The two substances had opposite effects on height and the concentration of PFOS interacted with child sex, suggesting a potential mechanism of action. Thomas Horvatits and colleagues from the University of Hamburg, Germany, investigated the potential accumulation of microplastics in the liver. In their proof-of-concept case series, they observed that six different microplastics were detectable in the livers of patients with cirrhosis, whereas none could be measured in individuals without underlying liver disease. Future studies are needed to understand whether this accumulation of microplastics is a cause or a consequence of tissue fibrosis. To conclude, we can only renew the pledge we made 1 year ago and encourage submissions of research articles, reviews, and commentaries along the intersection of environment and human health.

The important research articles published on these three topics should not overshadow the findings shared by our authors in other fields, such as oncology, allergy, neurology, digital health, genomics, and infectious diseases. We invite you to explore our archives (all articles are open access) to discover more. Finally, we would like to express our appreciation of the trust placed in the journal by our authors and of the hard work of the reviewers and advisory board members who shared their time and expertise with us. Without their support, the timely publication of exceptional science would not be possible. We are looking forward to new collaborations and exciting science in 2023!

